# Hemorrhagic Transformation in Ischemic Moyamoya Disease: Clinical Characteristics, Radiological Features, and Outcomes

**DOI:** 10.3389/fneur.2020.00517

**Published:** 2020-06-25

**Authors:** Junlin Lu, Zelin Li, Yuanli Zhao, Xiaolin Chen, Guangchao Shi, Jizong Zhao

**Affiliations:** ^1^Department of Neurosurgery, Beijing Tiantan Hospital, Capital Medical University, Beijing, China; ^2^China National Clinical Research Center for Neurological Diseases, Beijing, China; ^3^Center of Stroke, Beijing Institute for Brain Disorders, Beijing, China; ^4^Beijing Key Laboratory of Translational Medicine for Cerebrovascular Disease, Beijing, China; ^5^Beijing Translational Engineering Enter for 3D Printer in Clinical Neuroscience, Beijing, China; ^6^Department of Neurosurgery, Peking University International Hospital, Peking University, Beijing, China

**Keywords:** hemorrhage, hemorrhagic transformation, moyamoya disease, outcome, stroke

## Abstract

**Objective:** Hemorrhagic transformation (HT) in ischemic moyamoya disease (MMD), reasonably defined as hemorrhage events in patients with ischemic onset manifestation, leads to a poor outcome. This study aims to reveal factors associated with HT in patients with ischemic onset manifestation and to assess the outcome of these patients.

**Methods:** A total of 683 surgically managed patients with onset ischemic manifestation were included. The clinical variables of the HT and non-HT groups were compared, and risk factors were analyzed using logistic regression analysis. Recurrent stroke events (including hemorrhagic and ischemic) during the follow-up were documented. The cumulative incidence rate of stroke events was generated *via* Kaplan–Meier survival analysis. Outcomes were compared between HT and non-HT groups using propensity score analysis to account for between-group differences in baseline characteristics.

**Results:** Of 683 patients surgically treated in the overall cohort, 29 (4.3%) were classified as cases of HT. The majority manifestation of these patients was transient ischemic attack. Multivariate analysis showed that the normal cerebral perfusion according to the CT perfusion was identified as factors associated with HT [odds ratio (OR) 13.464, 95% CI 3.529–51.363, *P* < 0.001]. Patients who occurred HT had a worse outcome than patients without HT.

**Conclusions:** HT in adult ischemic MMD is a rare phenomenon, but it is strongly associated with increased disability rates and mortality. The normal cerebral perfusion is a possible risk factor associated with HT in adult ischemic MMD. Recognition of HT in adult ischemic MMD may contribute to an improved outcome.

## Introduction

Moyamoya disease (MMD) is a chronic occlusive-stenosis disorder at the terminal portion of the internal carotid artery, leading to intracranial ischemia and hemorrhage (1). The exact underlying mechanism between ischemic MMD and hemorrhagic MMD may be different and poorly understood. Although intracranial hemorrhage was less common than ischemic attack, it does contribute to the poor outcome in patients with MMD (2). Unlike most ischemic MMD patients who suffer from ischemic events, some patients with ischemic onset manifestation also developed intracranial hemorrhage, which we define as hemorrhagic transformation (HT). However, the clinical characteristics, radiological features, and outcomes of the ischemic MMD patients with HT remain unclear. At present, it is challenging to distinguish whether the patients will develop HT. As intracranial hemorrhage is hardly observed in pediatric MMD patients, in this study, we aim to explore the difference of clinical and radiological features between adult ischemic MMD patients with HT and without HT. Moreover, we studied the association of clinical outcomes and HT in adult ischemic MMD patients.

## Materials and Methods

### Patients and Materials

**Figure 1:**
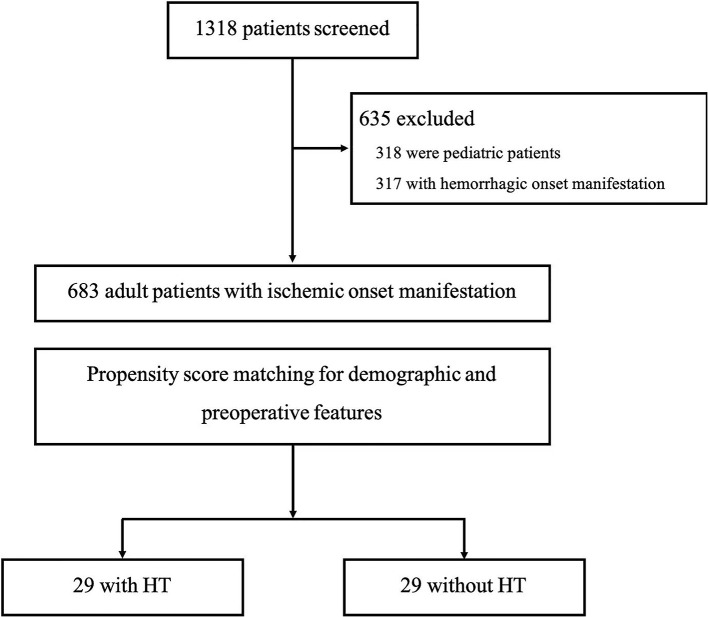
Flow diagram of study population.

The participants included in this study were from a multicenter cohort of Chinese MMD patients who had been treated between 2009 and 2018 (Figure 1). All patient's data were retrospectively reviewed, including clinical records and radiological data. The diagnosis of MMD was confirmed with digital subtraction angiography (DSA) and/or MR angiography based on the criteria of the Research Committee on Spontaneous Occlusion of the Circle of Willis (2012) (3). Pediatric and patients with hemorrhagic symptoms as the initial presentation and moyamoya syndrome caused by other systemic diseases were excluded.

Baseline clinical characteristics and imaging data were reviewed, such as age, sex, onset manifestation, past medical history, neurological status, and imaging findings. The onset manifestations were divided as follows: cerebral infarction, transient ischemic attack (TIA), headache, epilepsy, and asymptomatic. Suzuki stage was recorded as previously described (4). CT perfusion (CTP) was initiated 4 s after injection of a bolus of 40 ml of iobitridol (350 mg/ml, Xenetix; Guerbet, Aulnay-sous-Bois, France) at a rate of 5 ml/s into the antecubital vein (with a 20-gauge intravenous cannula) using a power injector. The acquisition parameters were as follows: 80 kVp, 150 mA, 0.28 s/rotation, 25 s CT data acquisition time, 5-mm section thickness, field matrix 512 × 512, and 40 images per section. The scan layers that contained basal ganglia were chosen in all patients. Cerebral perfusion assessment was performed by the new CTP staging system (pre-infarction staging system) proposed by our previous study (5). Neurological status was recorded using the modified Rankin Scale (mRS) score.

### Surgical Modalities

Our principles of the surgical strategies were as follows. Revascularization surgery was performed on the symptomatic and hemodynamically affected hemisphere. Patients usually reviewed 3–6 months after the first surgery. If the patient's symptoms were significantly relieved after the first surgery and the patient did not have symptoms that could be attributed to the contralateral hemisphere, operative treatment of the contralateral hemisphere was not considered. Otherwise, surgery on the contralateral hemisphere was performed. The surgical procedures of MMD could be divided into two categories in our population: indirect bypass and combined bypass (6).

### Follow-Up

Patients were followed up by clinical visit or telephone interviews at 3 and 6 months after surgery and annually thereafter. Doctors performing follow-up assessments were blinded to baseline information. Clinical outcomes including recurrent intracranial hemorrhage and ischemic stroke and neurological status were collected during follow-ups.

### Statistical Analysis

All analyses were conducted using IBM SPSS statistical software (version 26.0). The categorical variables are presented as counts (with percentages); continuous variables are presented as the means ± standard deviations. The chi-square test was used to compare categorical variables. The Mann–Whitney *U*-test was performed for ordinal variables. Logistic regression and proportional odds regression were used to generate odds ratios (ORs) and 95% confidence intervals (CIs). A logistic regression model was built to identify predictors of HT in ischemic MMD patients.

Propensity score matching was used to reduce imbalances in the baseline characteristics between patients with or without HT. The propensity scores for the development of HT in ischemic MMD were estimated with a logistic multivariate regression model containing demographic characteristics (age and sex), pre-operative angiopathy (Suzuki stage, posterior circulation involvement, and bilateral lesions), and clinical status (mRS score on admission, CTP stage, and previous revascularization surgery). Using the nearest-neighbor method without replacement for propensity score matching, pairs of patients were matched with a match tolerance of 0.02 and a ratio of 1:1. In the matched pairs, the outcomes of interest were stroke events (including intracranial hemorrhage and ischemic stroke), neurological function deterioration, disability-free rate, and mRS scores during follow-up. Cumulative risk of hemorrhage was estimated by the Kaplan–Meier product-limit method. A *P* value < 0.05 was considered to be statistically significant.

## Results

### Patient Characteristics

**Figure 2:**
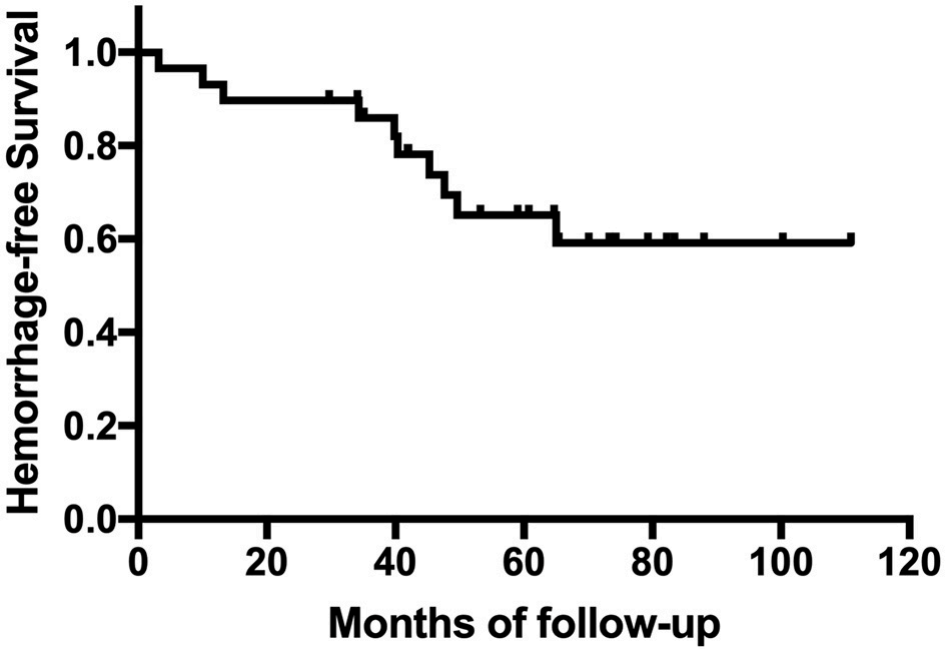
Kaplan–Meier curve for cumulative rates of hemorrhage for patients with hemorrhagic transformation (HT).

A total of 683 cases were included in this study (Figure 1). Baseline presentation and characteristics of the patient cohort are presented in Table 1. Of them, 29 patients developed HT. The occurrence of HT in adult ischemic MMD patients was 4.2%. The mean age of the patients with HT was 39.07 ± 10.39 years (range, 19~59 years). The female-to-male ratio was 1:1. The most common onset manifestation was TIA (18/29, 62.1%). Twenty-one patients (21/29, 72.4%) developed the HT before the revascularization surgery, 11 of them (TIA in 10 cases and infarction in 1 case) suffered an intracranial hemorrhage within the 3 months after the initial ischemic symptom. And eight patients (8/29, 28.6%) developed the HT after the revascularization surgery (mean time, 34.8 ± 19.8 months; range, 3~65 months). Intraventricular hemorrhage (IVH) and intracerebral hemorrhage (ICH) were the most frequent presentations observed on CT scans, accounting for 41.4% (12/29) and 37.9% (11/29), respectively. Besides, four patients were demonstrated ICH + IVH and two patients were demonstrated subarachnoid hemorrhage (SAH). The Suzuki stage of patients mostly were stage III and stage IV according to the Suzuki classification. All patients with HT underwent CTP. The measure of CTP was performed within 3 days after admission, and all patients were not in the acute stage of cerebral hemorrhage. Of them, five patients (5/29, 17.2%) with normal perfusion, zero patients (0.0%) in stage I, six patients (6/29, 20.7%) in stage II, 11 patients (11/29, 37.9%) in stage III, and seven patients (7/29, 24.1%) in stage IV. Kaplan–Meier curves for intracranial hemorrhage-free survival of patients with HT are shown in Figure 2.

**Table 1:** Baseline characteristics.

**Characteristic**	**Total (*n* = 683)**	**Hemorrhagic transformation**		***p*-value**
		**Present (*n* = 29)**	**Absent (*n* = 654)**	
Mean age, y	38.99 ± 9.92	39.07 ± 10.39	38.99 ± 9.91	0.967
Sex male/female	312/371	17/12	295/359	0.153
Onset manifestation				0.168
TIA	289 (42.3)	18 (62.1)	271 (41.4)	
Infarction	325 (47.6)	11 (37.9)	314 (48.0)	
Headache	49 (7.2)	0 (0.0)	49 (7.5)	
Epilepsy	12 (1.8)	0 (0.0)	12 (1.8)	
Asymptomatic	8 (1.2)	0 (0.0)	8 (1.2)	
mRS on admission				0.567
0	38 (5.6)	0 (0.0)	38 (5.8)	
1	452 (66.2)	19 (65.5)	433 (66.2)	
2	145 (21.2)	8 (27.6)	137 (20.9)	
3	36 (5.3)	2 (6.9)	34 (5.2)	
4	12 (1.8)	0 (0.0)	12 (1.8)	
Medical history				
Hypertension	238 (34.8)	8 (27.6)	230 (35.2)	0.402
Diabetes	79 (11.6)	3 (10.3)	76 (11.6)	0.833
Hyperlipemia	43 (6.3)	4 (13.8)	39 (6.0)	0.089
Smoking	94 (13.8)	5 (17.2)	89 (13.6)	0.578
Suzuki stage^*^				0.760
I	5 (0.9)	0 (0.0)	5 (0.9)	
II	31 (5.4)	2 (6.9)	29 (5.3)	
III	168 (29.2)	8 (27.6)	160 (29.3)	
IV	205 (35.7)	8 (27.6)	197 (36.1)	
V	122 (21.2)	7 (24.1)	115 (21.1)	
VI	44 (7.7)	4 (13.8)	40 (7.3)	
Posterior involvement^*^	177 (30.8)	13 (44.8)	164 (30.0)	0.093
Bilateral lesions	607 (88.9)	27 (93.1)	580 (88.7)	0.459
CTP stage				<0.001
Normal	15 (2.3)	5 (17.2)	10 (1.6)	
I	28 (4.3)	0 (0.0)	28 (4.5)	
II	189 (29.2)	6 (20.7)	183 (29.6)	
III	202 (31.2)	11 (37.9)	191 (30.9)	
IV	213 (32.9)	7 (24.1)	206 (33.3)	

*Values are numbers of cases (%) unless otherwise indicated. Mean values are presented with SDs*.

* *DSA was available in 575 patients*.

*^†^CT perfusion was available in 647 patients*.

### Risk Factors of Hemorrhagic Transformation

Univariable and multivariable ORs for the risk factors of HT are shown in Table 2. Univariate analysis showed that patients with normal cerebral perfusion status were more likely to develop HT. The multivariate analysis demonstrated that the normal cerebral perfusion status remains significantly associated with an increased risk of HT. Age, sex, hypertension, diabetes, hyperlipemia, smoking, TIA manifestation, Suzuki stage, posterior involvement, bilateral lesions, and previous revascularization surgery history were not associated with any increased risk of HT in the analysis (*P* > 0.05).

**Table 2:** Logistic regression analysis for hemorrhagic transformation in adult ischemic MMD.

**Characteristic**	**Univariate analyses**			**Multivariate analyses**		
	**OR**	**95% CI**	***P*-value**	**OR**	**95% CI**	***P*-value**
Mean age, y	1.001	0.964–1.039	0.967	1.008	0.963–1.506	0.720
Male	1.724	0.810–3.668	0.157	1.878	0.785–4.494	0.157
Hypertension	0.702	0.306–1.611	0.404	0.530	0.200–1.408	0.203
Diabetes	0.878	0.259–2.969	0.834	1.010	0.243–4.195	0.989
Hyperlipemia	2.523	0.837–7.610	0.100	3.437	0.880–13.417	0.076
Smoking	1.323	0.492–3.556	0.580	0.854	0.259–2.817	0.796
TIA manifestation	2.313	1.075–4.975	0.032	2.150	0.936–4.939	0.071
Suzuki stage	1.170	0.820–1.670	0.387	1.111	0.743–1.663	0.608
Posterior involvement	1.893	0.890–4.024	0.097	1.902	0.830–4.361	0.129
Bilateral lesions	1.722	0.401–7.391	0.464	1.406	0.285–6.935	0.676
CTP stage						
Normal	REF	REF	REF	REF	REF	REF
I	<0.001	–	0.998	<0.001	–	0.998
II	0.066	0.017–0.252	<0.001	0.057	0.012–0.261	<0.001
III	0.115	0.034–0.395	0.001	0.132	0.031–0.558	0.006
IV	0.068	0.018–0.252	<0.001	0.057	0.013–0.254	<0.001
Previous revascularization	1.512	0.681–3.356	0.309	1.406	0.572–3.460	0.458

### Hemorrhagic Transformation and Long-Term Clinical Outcomes

**Figure 3:**
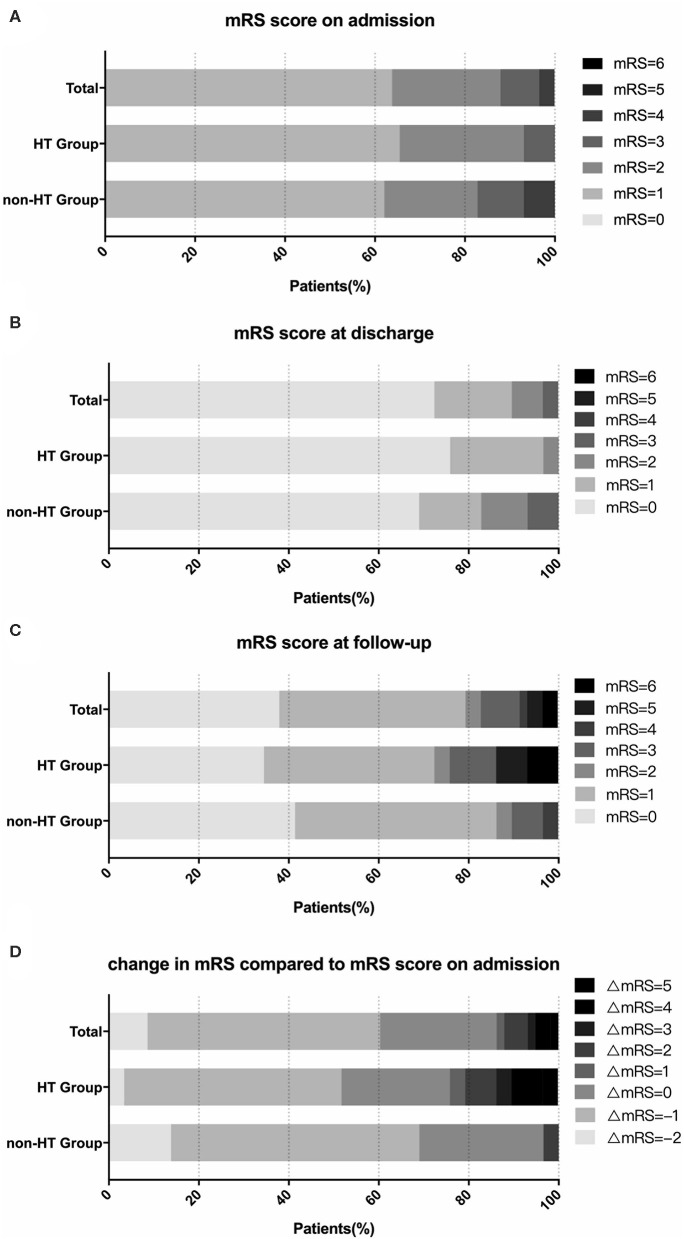
Change in modified Rankin Scale (mRS) scores in the propensity score-matched cohort. The graphs show the proportions of patients stratified by mRS score. There was no statistically significant difference in mRS scores on admission **(A)**, at discharge **(B)**, and at follow-up **(C)** between the hemorrhagic transformation (HT) and non-HT groups. Compared to their neurological status on admission, the mRS score at follow-up showed a deterioration in 20.7% of patients in the HT group but 0% of the non-HT group **(D)**.

Within the overall propensity score-matched cohort of 58 patients, the two groups were compared with each other to verify that no significant differences were present in baseline characteristics between these two groups after the propensity score matching (Table 3). Outcomes of patients in the propensity score-matched cases are shown in Table 4. During the follow-up period (mean time, 71.9 ± 29.0 months; range, 12~116 months), 15 stroke events (including 10 intracranial hemorrhages and five ischemic strokes) were observed, two patients experienced both intracranial hemorrhage and ischemic stroke. One of them experienced multiple hemorrhage events, which contributed to a poor outcome. There was a significant difference in the mRS score between these two groups at follow-up (*P* = 0.047; Figure 3). Forty-six patients (46/58, 79.3%) recovered without disability at the final follow-up. More patients in the HT group were disabled or dead (mRS score 3~6) than in the non-HT group (7/29, 24.1 vs. 3/29, 10.3%, *P* = 0.086). In comparison to the mRS score on admission, the mRS score at the last follow-up showed improvement of 1–2 points in 35 cases (35/58, 60.3%), remained unchanged in 15 cases (15/58, 25.9%), and eight patients (8/58, 13.8%) had worse neurological functions. Follow-up mRS scores showed deterioration (compared to the scores on admission) in seven patients (7/29, 24.1%) in the HT group but only one patient (1/29, 3.4%) in the non-HT group (*P* = 0.05; Figure 3). However, it should also be noted that five patients (5/29, 17.2%) in the non-HT group had poor neurological functions on admission (mRS score 3~4), in contrast to one patient (1/29, 3.4%) in the HT group (Figure 3).

**Table 3:** Baseline characters of patients in the propensity score–matched cases.

**Characteristic**	**Total (*n* = 58)**	**Hemorrhagic transformation**		***P*-value**
		**Present (*n* = 29)**	**Absent (*n* = 29)**	
Mean age, y	38.88 ± 10.16	39.07 ± 10.39	38.69 ± 10.10	0.888
Sex male/female	38/20	17/12	21/8	0.269
Onset manifestation				0.188
TIA	31 (53.4)	18 (62.1)	13 (44.8)	
Infarction	27 (46.6)	11 (37.9)	16 (55.2)	
mRS on admission				0.335
1	38 (65.5)	19 (65.5)	18 (62.1)	
2	14 (24.1)	8 (27.6)	6 (20.7)	
3	4 (6.9)	2 (6.9)	3 (10.3)	
4	2 (3.4)	0 (0.0)	2 (6.9)	
Medical history				
Hypertension	16 (27.6)	8 (27.6)	8 (27.6)	1.000
Diabetes	7 (12.1)	3 (10.3)	4(13.8)	0.687
Hyperlipemia	8 (13.8)	4 (13.8)	4 (13.8)	1.000
Smoking	9 (15.5)	5 (17.2)	4 (13.8)	0.717
Suzuki stage				0.729
I	0 (0.0)	0 (0.0)	0 (0.0)	
II	3 (5.2)	2 (6.9)	1 (3.4)	
III	13 (22.4)	8 (27.6)	5 (17.2)	
IV	20 (34.5)	8 (27.6)	12 (41.4)	
V	15 (25.9)	7 (24.1)	8 (27.6)	
VI	7 (12.1)	4 (13.8)	3 (10.3)	
Posterior involvement	21 (36.2)	13 (44.8)	8 (27.6)	0.172
Bilateral lesions	53 (91.4)	27 (93.1)	26 (89.7)	0.640
CTP stage				0.220
Normal	7 (12.1)	5 (17.2)	2 (6.9)	
I	0 (0.0)	0 (0.0)	0 (0.0)	
II	19 (32.8)	6 (20.7)	13 (44.8)	
III	19 (32.8)	11 (37.9)	8 (27.6)	
IV	13 (24.1)	7 (24.1)	6 (20.7)	

**Table 4:** Outcomes of patients in the propensity score–matched cases.

**Outcomes**	**Total (*n* = 58)**	**HT Group (*n* = 29)**	**non-HT Group (*n* = 29)**	***P*-value**	**OR (95% CI)**	**Adjusted *P*-value**
mRS score at discharge				0.321^†^	0.752 (0.198–2.861)^‡^	0.676
1	42 (72.4)	22 (75.9)	20 (69.0)			
2	10 (17.2)	6 (20.7)	4 (13.8)			
3	4 (6.9)	1 (3.4)	3 (10.3)			
4	2 (3.4)	0 (0.0)	2 (6.9)			
Stroke event during follow-up	13 (22.4)	10 (34.5)	3 (10.3)	0.028^*^	4.561(1.103–18.859)	
Hemorrhage during follow-up	10 (17.2)	10 (34.5)	0 (0.0)	0.001^*^	1.526 (1.172–1.988)	
Infarction during follow-up	5 (10.3)	2 (6.9)	3 (8.6)	0.640^*^	0.642 (0.099–4.159)	
mRS score at follow-up				0.476^†^	1.693 (1.007–2.846)	0.047
0	22 (37.9)	10 (34.5)	12 (41.4)			
1	24 (41.4)	11 (37.9)	13 (44.8)			
2	2 (3.4)	1 (3.4)	1 (3.4)			
3	5 (8.6)	3 (10.3)	2 (6.9)			
4	1 (1.7)	0 (0.0)	1 (3.4)			
5	2 (3.4)	2 (6.9)	0 (0.0)			
6	2 (3.4)	2 (6.9)	0 (0.0)			
Disability at follow-up (mRS > 2)	10 (17.2)	7 (24.1)	3 (10.3)	0.164^*^	4.355 (0.812–23.367)	0.086
Neurological function deterioration	8 (13.8)	7 (24.1)	1 (3.4)	0.022^*^	8.696 (0.986–76.722)	0.050

*Values are numbers of cases (%) unless otherwise indicated. Mean values are presented with SDs*.

* *McNemar test*.

† *Marginal homogeneity test*.

‡ *Adjusted for mRS score on admission*.

## Discussion

There were mainly two typical manifestations in MMD patients: intracranial hemorrhage, which was commonly observed in adults, and ischemic stroke, which was mostly observed in pediatrics. Ischemic manifestations were more common than hemorrhagic manifestations (7, 8). MMD manifested as ischemia or hemorrhage at the initial attack may differ greatly in many aspects such as pathological features, disease progression, and prognosis (1, 9). The exact cause for hemorrhage in MMD has not been fully elucidated. Previous studies have revealed that the rupture of microaneurysms in the collateral vessels at the base of the skull was related to the hemorrhagic presentation in patients with MMD (10). The most common adverse event which contributed to a poor outcome in patients manifested as ischemia was recurrent ischemic stroke, while rebleeding plays an important role in patients with hemorrhagic manifestation. Recently, a long follow-up study reported that the average annual ischemic stroke incidence in patients with hemorrhagic manifestation was only 0.3% (11). However, there were few reports about intracranial hemorrhage in patients manifested as ischemia, considering that intracranial hemorrhage contributed to higher mortality than ischemic events in MMD patients (12, 13). This large cohort study extends our knowledge of the clinical characteristics, radiological features, and long-term outcome of adult ischemic patients with HT.

In this study, we found that the incidence of HT in adult ischemic MMD patients was 4.2%. Previous studies reported that HT occurs in as many as 10–40% of patients with ischemic stroke (14). The use of alteplase and the brain–blood barrier disruption might contribute to HT (15, 16). However, in our cohort, 11 patients developed HT within 3 months after the initial ischemic manifestation, while 18 patients experienced ischemic strokes or TIAs for more than 2 years. The underlying mechanism of HT in patients with MMD might differ from those with acute ischemic stroke. In addition, TIA was the most common symptom before intracranial hemorrhage in this cohort (18/29, 62.1%), and univariate analysis confirmed the significant association between TIA manifestation and incidence of HT (OR 2.313, 95% CI 1.075–4.975, *P* = 0.032). However, multivariate analysis showed no significance. It should also be noted that eight of them developed HT after the revascularization surgery. We suggest that adult ischemic MMD patients take 100 mg of aspirin (The Bayer Company) daily for antiplatelet therapy. Although our previous study showed that aspirin might not increase the risk of hemorrhages in adult ischemic MMD patients (17), whether aspirin is safe for patients with the potential of HT remains uncertain. The outcome of patients who takes aspirin may be worse when developing an intracranial hemorrhage than those who do not. In this study, we observed that patients who occurred HT after the revascularization surgery have a worse neurological function at the last follow-up than patients occurred HT before the revascularization surgery (mRS score 2.75 ± 2.32 vs. 1.05 ± 1.47, *P* = 0.025). Moreover, among 29 patients occurred HT, eight of these occurred HT after the surgery (six direct revascularization vs. two indirect revascularization). Patients with direct revascularization have a higher percentage of HT. The vessels of MMD patients might be abnormal vasodilation due to the chronic hypoperfusion. The immediate increase of the blood flow after the direct revascularization might increase the risk of hemorrhage.

The determination of the HT risk is essential to provide information for appropriate management for adult ischemic MMD. At present, various factors were found to be associated with intracranial hemorrhage in MMD. Kikuta et al. (18) reported the presence of multiple microbleeds on magnetic resonance imaging to be an independent predictor of intracranial hemorrhage. Liu et al. (19) found that the involvement of the posterior circulation was associated with the hemorrhage in MMD. Meanwhile, other factors such as smoking and hypertension have already been considered as risk factors for hemorrhagic stroke (20–22). However, in this study, univariable analysis revealed that posterior circulation involvement, smoking, and hypertension were not significant predictors of HT.

Furthermore, the results of CTP demonstrated that patients with HT had a higher proportion of normal cerebral perfusion than patients without HT (5/29, 17.2 vs. 10/654, 1.6%, *P* < 0.001). Interestingly, only 2.3% of patients in this cohort had normal cerebral perfusion, yet nearly more than half of them occurred HT. Multivariate analysis confirmed that normal cerebral perfusion status was significantly associated with HT (OR 13.464, 95% CI 3.529–51.363, *P* < 0.001). It should be noted that the patients were mostly in stage III and stage IV according to the Suzuki classification. In order to maintain a normal perfusion status, these patients are usually accompanied with good collateral circulation compensations even some of which were regarded as dangerous collateral vessels and closely related to hemorrhagic stroke recurrence in adult hemorrhagic MMD, while the rupture of these vasodilated abnormal collateral vessels might contribute to intracranial hemorrhage (23). In this study, most patients presented with IVH and ICH (41.4 and 37.9%, respectively), only two patients presented with SAH, which represent the typical presentation of the ruptured collateral vessels in MMD. Therefore, it might provide some support for our hypothesis.

In propensity score-matched cases, we have shown that the mRS score at the last follow-up was significantly different between patients with HT and without HT (*P* = 0.047). The severe disability and mortality of patients with HT were higher than that of patients without HT, while it did not have a significant difference (*P* = 0.086). We also observed that more patients observed with neurological function deterioration were in the HT group (*P* = 0.05), but it might be because some patients developed HT during the follow-up period which caused neurological function deterioration. As aforementioned, we suggest adult ischemic MMD patients take aspirin for antiplatelet therapy. Therefore, we advise that the time period of taking aspirin should be more accurate in these patients to decrease the incidence and mortality of hemorrhage events.

There are study limitations that need to be addressed for accurate interpretation of our data. In this study, only adult ischemic patients treated surgically were included, and this introduced selection bias as patients with less severe MMD disease might have been treated conservatively and were excluded; therefore, the conclusion drawn may not be generalizable to all patients with MMD. Although cerebral CT or magnetic resonance imaging scans have been performed at the point of new symptom manifestations during follow-up, DSA was not routinely performed, and the data are, therefore, not available in some of the patients; thus, limited radiographic evidence of disease progression must be noted. In addition, similar to all retrospective studies, this is a multicenter study over a time span of ≈10 years, and not all patients were followed up on a regular basis. This may render the results prone to potential attrition biases.

## Conclusions

In summary, HT in adult ischemic MMD is a rare phenomenon. Our data support that normal cerebral perfusion is a possible risk factor associated with HT in adult ischemic MMD. HT was strongly associated with increased disability rates and mortality. The concept of HT in adult ischemic MMD may contribute to further improvement in the outcome of MMD as a result of appropriate antiplatelet management arising from HT risk stratification.

## Data Availability Statement

The datasets generated for this study are available on request to the corresponding author.

## Ethics Statement

The studies involving human participants were reviewed and approved by IRB of Beijing Tiantan Hospital, Capital Medical University. The patients/participants provided their written informed consent to participate in this study.

## Author Contributions

JL designed the study, drafted the manuscript, performed data collection and data analysis, contributed to the discussion, and agreed to be accountable for all aspects of the work in ensuring that questions related to the accuracy or integrity of any part of the work are appropriately investigated and resolved. ZL performed data collection and contributed to the discussion. XC and YZ contributed to the discussion and edited the manuscript. GS performed data collection. JZ provided approval for publication of the content. All authors contributed to the article and approved the submitted version.

## Conflict of Interest

The authors declare that the research was conducted in the absence of any commercial or financial relationships that could be construed as a potential conflict of interest.
